# Diet-microbiota associations in gastrointestinal research: a systematic review

**DOI:** 10.1080/19490976.2024.2350785

**Published:** 2024-05-09

**Authors:** Kerith Duncanson, Georgina Williams, Emily C. Hoedt, Clare E. Collins, Simon Keely, Nicholas J. Talley

**Affiliations:** a NHMRC Centre of Research Excellence in Digestive Health, University of Newcastle, Newcastle, NSW, Australia; b Immune Health Program, Hunter Medical Research Institute, New Lambton Heights, NSW, Australia; c School of Medicine & Public Health, College of Health, Medicine and Wellbeing, University of Newcastle, Newcastle, NSW, Australia; d School of Biomedical Sciences & Pharmacy, College of Health, Medicine and Wellbeing, University of Newcastle, Newcastle, NSW, Australia

**Keywords:** Microbiota, microbiome, diet, dietary assessment, diet-microbiota correlation

## Abstract

Interactions between diet and gastrointestinal microbiota influence health status and outcomes. Evaluating these relationships requires accurate quantification of dietary variables relevant to microbial metabolism, however current dietary assessment methods focus on dietary components relevant to human digestion only. The aim of this study was to synthesize research on foods and nutrients that influence human gut microbiota and thereby identify knowledge gaps to inform dietary assessment advancements toward better understanding of diet–microbiota interactions. Thirty-eight systematic reviews and 106 primary studies reported on human diet-microbiota associations. Dietary factors altering colonic microbiota included dietary patterns, macronutrients, micronutrients, bioactive compounds, and food additives. Reported diet-microbiota associations were dominated by routinely analyzed nutrients, which are absorbed from the small intestine but analyzed for correlation to stool microbiota. Dietary derived microbiota-relevant nutrients are more challenging to quantify and underrepresented in included studies. This evidence synthesis highlights advancements needed, including opportunities for expansion of food composition databases to include microbiota-relevant data, particularly for human intervention studies. These advances in dietary assessment methodology will facilitate translation of microbiota-specific nutrition therapy to practice.

## Introduction

The gastrointestinal (GI) microbiota is increasingly implicated in maintaining good overall health and is important in both preventing and managing GI and extraintestinal diseases.^1,2^ Dietary intake affects the colonization, abundance, diversity, and metabolic influences of the GI microbiota.^3^ Methods used to assess and analyze human dietary intake focus on nutrients that are specific to human digestion, absorption and metabolism,^4^ not to human GI microbial metabolism, which means the extent and nature of these influences are currently not well characterized.^3,5^

Knowledge of GI microbes increased exponentially when gene sequencing superseded microbial culture. Assessment of the abundance and diversity of microbial taxa has become more detailed with use of next-generation sequencing technologies such as 16S rRNA amplicon gene sequencing and metagenomic shotgun sequencing (MGS). With the reduction of cost for MGS, this is fast becoming the preferred approach to microbiota profiling as it captures the taxonomic composition in a non-biased manner and reports the microbial metabolic functional properties.^6,7^ These technological advances have revealed that gut-derived metabolites are intermediaries between the gut microbiota and host metabolism and that differential responses to diet between individuals may relate to the interplay between microbes, the food metabolome,^8,9^ and human host factors, including their genetic profile and gene expression.^10,11^

Improvements in sample collection methods have further enhanced specificity and sensitivity of microbial analysis, allowing for identification of microbial niches along the GI tract. For example, duodenal mucosal samples can now be extracted using aseptic techniques, facilitating accurate analysis of mucosa-associated microbiota in this important part of the digestive tract.^12^ Despite these advances, mechanistic microbiome studies are challenging to conduct and prone to error due to the complexity of the gut microbiota, and intra- and inter-individual variability.^5^

The vastly expanded horizon of understanding regarding gut microbiota afforded by gene sequencing is likely to revolutionize research into, and understanding of digestion, absorption, and metabolism of nutrients in food. Nutrients and compounds in foods that have low bioavailability could ‘feed’ the colonic microbiota. Additives that are generally recognized as safe for human health have not been adequately assessed in relation to microbiota interactions.^13^

Consolidation of diet-microbiota data for meta-analysis or meaningful synthesis is limited by the variability in microbiological samples, sampling techniques, dietary intake data, study designs and outcome measures in studies conducted to date. Systematic reviews consistently report limitations to dietary assessment methods for analyzing microbiota-relevant diet,^14^ foods and nutrients.^15–18^ While some data reported in these reviews are from human studies, research in the microbiome field continues to be highly reliant on animal data,^19,20^ which may not be well distinguished from human data in some reviews.

To progress toward the goal of diet-focused microbiota manipulation for health status optimization, the specificity and sensitivity of dietary assessment and analysis methods used in diet-microbiome research needs to improve. Such progress is reliant on better differentiation and delineation of human from animal study findings, increased ability to discern correlation or association from causation^21^ and increased scrutiny of what nutrients are accessible to the microbiota in respective parts of the GI tract. As the purported effects of microbiota on human metabolism relate to the metabolites produced by those microorganisms, there also needs to be mechanisms for dietary, microbiota, and metabolomic data to be triangulated in analysis.

Therefore, the aim of this systematic review was to determine which foods, food components and whole foods consumed by humans, influence gut microbiota composition, and to identify gaps and opportunities for more relevant dietary assessment in diet-microbiome research.

## Results

Of 241 papers retrieved, 38 systematic reviews,^13–18,22–52^ and 106 primary studies^53–159^ reported on diet-microbiome associations in humans or in-vitro laboratory studies with human fecal samples or human models, and were included in this review. The dietary aspects studies were categorized and reported as dietary patterns, dietary carbohydrate, protein, fat, micronutrients, bioactive food compounds, food additives, and individual foods.

### Dietary patterns and the microbiota

Six systematic reviews^13,14,22–24,43^ and 10 primary studies^53–61,137^ reported on associations between dietary patterns and microbiota (Supplementary Table S1). Of these, four systematic reviews^13,22–24^ and six primary studies^53–57,137^ reported on associations between vegan or vegetarian, ‘healthy’, ‘plant-based’, or ‘Mediterranean’ dietary patterns and microbiota, with up to six microbial species associations reported in one study.^54^ One systematic review^14^ and four primary studies^54,58–60^ investigated the effect of ‘western’, less healthy’, or ‘animal-based’ dietary patterns on microbiota, and reported associations with up to seven associations across taxonomic levels. Two systematic reviews^14,43^ and two primary studies^60,61^ reported on the effects of alterations in total energy intake on microbiota, with up to eight bacterial associations reported.^60^ Studies that reported on associations between dietary patterns and intestinal microbiota differentiated the total diet dichotomously as either ‘plant’ or ‘animal’ based, vegan/vegetarian or omnivorous or as a ‘healthy’ or ‘unhealthy’ diet pattern based on total energy and macronutrient distribution.

Criteria for delineating specific dietary patterns were heterogenous in both review papers^14,22–24^ and primary studies.^53–55,58–60^ In studies that assessed or reviewed vegan or vegetarian diets compared to an omnivorous diet or ‘animal-based’ diet, inclusion criteria often included a predetermined period of adherence to the diet under study, which was either self-reported by participants (retrospective and usually longer duration of up to one year) or prospectively prescribed or provided as part of the intervention (usually 1 to 3 months).^22^ For comparison of Mediterranean style or ‘healthy’ with a ‘Western’ or ‘less healthy’ dietary pattern, a classification tool was used to rank or score the diet, before, associations with microbiota or microbiome were analyzed statistically. Some studies used validated dietary assessment tools, while others involved more arbitrary classification systems or post-hoc delineation to categorize dietary intakes as ‘healthy’ or ‘unhealthy’ based on study-specific criteria.^57^

In reviews and primary studies included, dietary patterns were compared with species-level microbial abundance,^22,23,54,58–60^ microbial diversity^54^ and microbial enterotype.^14,53^ ‘Healthy’, ‘plant-based’ and ‘Mediterranean’ dietary patterns were generally associated with higher or increased Bacteroidetes (phylum),^13,22^ Firmicutes (phylum),^13,54^ *Prevotella*,^13,22^ *Roseburia*,^54,57^ *Faecalibacterium prausnitzii*,^54^ *Bifidobacteria*, and *Haemophilus*.^55^ *Xylanibacter*, *Prevotella*, *Butyrivibrio* and *Treponemaare* genera were found to be exclusive to people on a plant-based diet in one study.^56^ One review reported an association between the Mediterranean diet and microbial metabolite ((short chain fatty acid (SCFA)) production^13^ and other studies reported the functional attributes of specific microbes that were relevant to the dietary pattern.^54,58–60^ A ‘Western’, ‘animal-based’ or ‘less healthy’ dietary pattern generally reported lower abundance of the same microbiota,^58–60^ often because of the dichotomous, relative nature of analysis. These dietary patterns were specifically associated with increased genera *Bacteroides*,^14,58^ *Clostridium*,^54^ *Alistipes*^58^ and *Bilophila wadsworthia*.^58^

Studies that reported on associations between total energy (Calorie or kilojoule) intake and GI microbiota varied in the primary and secondary outcomes assessed. One systematic review focused on interventions that achieved either increased or decreased energy intake while maintaining macronutrient ratios.^14^ One review investigated the impact of a very low energy, ultra-processed diet^43^ and another compared microbiota outcomes before and after energy restriction with modified macronutrient ratios.^60^ Despite these differences, lower (or reducing) energy intake compared to higher (or increasing) total energy intake generally resulted in increased abundance of Bacteroidetes,^14,43^ decreased Firmicutes^43,60^ and increased GI microbial diversity.^14^

### Dietary carbohydrates, fibres and the microbiota

Twelve systematic reviews^14–17,25–30,42,45^ and 32 primary studies^44,58–61,65,74–95,135,136,140,150^ reported associations between microbiota and either total digestible carbohydrates, total fibers, specific digestible carbohydrates or specific fiber types, with up to 52 associations reported across six studies reported in one review^25^ (Supplementary Table S2).

Carbohydrate constituents of food reported in papers included in this review included digestible carbohydrate intake (total, high/low diet, saccharide type), fiber amount (total fiber or as higher/lower fiber), human enzyme digestibility (level of processing, resistant starch), availability to microbiota (reported as prebiotic, total fermentable carbohydrate, total fructans, fructooligosaccharides, galactooligosaccharides, oligofructose, raffinose, arabinoxylose, trehalose, polydextrose, sialic acid, resistant starch), fermentable oligosaccharide, disaccharide, monosaccharide and polyol (FODMAP) content (total, low FODMAP diet, fructose, lactose, polyols), by cereal grain amount and type (barley, rye, oats, wheat), grain fiber type (wheat, rye and lupine fiber, insoluble fiber) or grain processing (total wholegrains, intact cereal grains, grain size).

Carbohydrate or fiber-microbiota associations reported were generally as expected, with higher dietary fiber associated with higher or increased beneficial microbes^14–17,25–27,30,42,45,60,61,65,74,76–84,88–91,136,140^ and the influence of carbohydrate varying depending on the food source and carbohydrate type.^17,28,29,58–60,75,82,85–87,92–95,135,150^

A systematic review that included 34 (intervention and cross-sectional) studies reported that high dietary fiber intake was associated with greater gut bacteria diversity and increased Firmicutes, Proteobacteria (phylum), *Prevotella*, *Bifidobacterium* and *Lactobacillus* genera, and increased and decreased Actinobacteria (phylum).^14^ Reported associations between fiber and Bacteroidetes varied between studies, depending on whether phylum or species level data was measured, and the study design or granularity of dietary intake analysis.

One systematic review^14^ reported that resistant starch intake (from supplementation or specific formulated food sources) increased *Eubacteria* and *Ruminococcus*. One review reported that higher (or addition of) resistant starch increased *Bifidobacteria*.^17^ Interestingly, a lack of *Ruminococcus bromii* at baseline was associated with lower resistant starch digestion of 20–30% compared to 100% in participants with *Ruminococcus bromii* in their gut microbiota^14^ indicating the importance of baseline microbiota assessment and consideration of intra-individual responses. The type of resistant starch being investigated influenced the microbial response, although few studies have compared microbial responses to all resistant starch types in the same study sample^78^ and study populations also differ between studies.^60,61^

By definition, prebiotic fibers promote proliferation of beneficial bacteria or a beneficial microbial profile. Prebiotic fibers can be intrinsic to foods or consumed as supplements and were reported in studies in various ways. Total prebiotic fiber (intrinsic and supplemental) intake was reported together in some studies and separately in others, while other studies reported associations between individual prebiotic fibers and bacteria separately. Total prebiotic fiber, specific prebiotic fiber-rich foods, or supplements and total or specific whole grain foods or supplements were reported to have similar effects on microbiota, despite diverse study designs. Commonly reported associations included increased microbial diversity and abundance, increased Firmicutes, Ruminococcaceae (family), *Bacteroides*, *Bifidobacterium*, *Lactobacillus*, *Anaerostipes*, *F. prausnitzii, Prevotella*, *Clostridium* spp. and fecal butyrate,^14,15,17,26–29,44,85,86,90,91^ lower relative abundance of Bacteroidetes, *Escherichia coli*^26,28,88^ and inconsistent results reported for Clostridia class.^15,26,28,91^

Dietary approaches to the management of functional GI disorders involve manipulation of fermentable carbohydrate intake to alleviate GI pain and bowel symptoms. For example, the low FODMAP diet is an evidence-based dietary management approach for IBS, and its impact on bacterial fermentation has been investigated in both primary studies and systematic reviews.^16,79^ One systematic review^16^ and a primary study^79^ that involved healthy individuals and people with IBS reported that a high FODMAP (compared to low FODMAP) diet resulted in microbiota changes that were comparable with those reported for supplementation with other prebiotic fibers. Associations with higher FODMAP intake included higher microbial abundance, fecal volume, gene richness, Ruminococcaceae (family), Lachnospiraceae (family), *Lactobacillus* (genus), *Clostridium* cluster XIVa (spp.), *Akkermansia muciniphila* (sp.), *F. prausnitzii* (sp.), *Bilophila wadsworthia* (sp.) and lower Clostridia (class) and *Enterococcus* (genus).^16,79^ A low FODMAP diet has also been associated with increased Clostridiales family XIII, *Porphyromonas* IV^16^ (genus), *Bacteroides*,^45^ Bacteroidetes^65^ and lower Propionibacteriaceae (family), *Bifidobacterium*, Clostridium cluster IV (spp.) and *Ruminococcus torques*.^16,30,79^ A recent systematic review and meta-analysis of low FODMAP diets and microbiota outcomes in IBS reported that lower relative abundance of *Actinobacteria* (4 studies) was the only consistently reported association, with variable outcomes at phylum and genus level for all other reported microbes and SCFA.^45^

### Dietary protein, amino acids and the microbiota

Five reviews^14,31,32,40,41^ and 20 primary studies^7,46,62–73,138,139,154–157^ reported on associations between microbiota and total dietary proteins, specific peptides or amino acid, with up to 14 associations across taxonomic levels reported^62^ (Supplementary Table S3).

Reported associations between dietary protein and microbiota range from very general bacterial enterotype and total dietary protein to highly specific bacterial species and individual amino acids. Studies in this review indicated that increased or higher total dietary protein intake is usually associated with less beneficial bacterial taxonomic and metabolite profiles,^14^ although it is not possible to differentiate the effect of protein separately from the difference or change in total energy or the proportion of dietary fat or carbohydrate (and associated fiber).

Across the two reviews and four primary studies that reported on microbiota and dietary gluten intake, associations with 28 different bacterial species were reported. There were few consistent findings between studies, most likely due to the wide range of study designs. Inclusion criteria ranged from gluten-free to ‘low’ versus ‘high’ gluten intake, and study populations ranged from children to adults with or without celiac disease, IBS or non-celiac gluten sensitivity (NCGS), and analysis of samples from saliva, duodenal tissue or stool.^32,62–66^

Dietary intake of individual amino acids is not routinely assessed as part of nutrient analysis of total diet intake. One study reported reduced Firmicutes:Bacteroidetes following L-glutamine supplementation.^70^ One study each reported on microbial metabolism of proline,^71^ sulfur amino acids,^72^ biogenic amines^73^ and L-carnitine.^14^

### Dietary fat and the microbiota

Two systematic reviews^14,18^ and 11 primary studies^20,74,96–103,148^ reported associations between the microbiota and total, saturated or unsaturated fats, with up to four bacterial associations in primary studies^74,101–103^ (Supplementary Table S4).

Assessment of the absolute effect of total fat, dietary fat profile and individual fatty acids on microbiota in humans is complex. Altering total fat intake influences total energy intake or macronutrient proportions, as well as bile production, which all independently influence microbiota.^43,60,160^ Overall, data in this review indicate that a high or increased intake of total fat and saturated fatty acids have a consistently negative effect on microbiota richness and diversity.^14,18^

Studies in this review reported high total fat intakes as being associated with higher or increased Bacteroidetes, Actinobacteria, *Alistipes*, and lower or decreased relative abundance of Firmicutes, Proteobacteria, *Bifidobacteria*, butyrate and total SCFAs,^14^ with saturated fats specifically linked to an unfavorable gut microbiota profile,^18^ increased *Bacteroides*, *Bilophila* (genus) and *F. prausnitzii*.^3,97^ One whole food-based dietary intervention reported 57 genus-level taxon differences between high saturated fat and a high MUFA diet.^99^ The same study suggested that high animal fat intake for 1 month was associated with a *Bacteroides* enterotype.^99^

Studies investigating the influence of dietary fats on microbiota did not consistently report similar or opposite effects of unsaturated fats on microbiota compared to saturated fats. Diets high in unsaturated fat and manipulation of unsaturated fatty acids had variable but generally less detrimental influences on relative microbial abundance, microbial richness or diversity^14,18^ (Supplementary Table S4).

### Micronutrients and the microbiota

Four reviews^14,33–35^ and one primary study^104^ reported on a range of direct and indirect associations between micronutrients and gut microbes (Supplementary Table S5). Some microbes, such as Eubacterium rectale and Porphyromonas gingivalis (sp) are reported to be dependent on vitamin B6 for enzymatic activities.^14^ Vitamin B12 intake and supplementation have been associated with gut microbiome diversity, relative abundance, functional capacity and SCFA production.^33^ Altered gut microbiota have also been reported following vitamin D supplementation.^34,35^

In relation to dietary or supplemental mineral intake, Clostridium (spp.) and fecal SCFAs were increased following administration of phosphorous and calcium in one study.^104^ Reduced Lactobacilli (g) has been reported in iron-deficiency anemia.^14^

In addition to some gut microbes being micronutrient dependent, microbes may also be involved in micronutrient production. Gut microbiota are involved in synthesis of b-carotene, vitamin B6 and folate, with vitamin B12 specifically reported as being synthesized by Propionibacterium freudenreichii (sp), Listeria innocua (sp) and Lactobacillus reuteri (sp).^14^ One review reported that Bifidobacterium (g) used resistant starch to produce folate.^14^

### Bioactive food compounds and the microbiota

Eight reviews^14,27,30,36,37^ reported phytochemical-microbiota associations (Supplementary Table S6). The phytochemicals that were most commonly reported to be associated with gut microbiota in this review were dietary fibers (see carbohydrate section), vitamin and minerals (see micronutrients section) and polyphenols, a heterogeneous collection of plant-derived compounds, with the structural unit of hydroxylated aromatic or phenol rings in common.^161^ Polyphenols were reported to influence gut microbiota to a similar extent to oligosaccharide prebiotics,^36^ with evidence from systematic reviews of human trials, intervention studies and some preclinical studies supporting this strong relationship.

Polyphenol consumption positively influenced gut microbiota, with bifidogenic effects reported in three systematic reviews^14,36,37^ and higher or increased *Lactobacillus* in two systematic reviews.^14,36^ Other bacteria reported to be higher or increase in relation to polyphenol intakes included Ruminococcaceae, Lachnospiraceae (family), *Lachnospira* (genus), *Anaerostipes*, *Bacteroides*, *Roseburia* (genus), *Prevotella*, *Enterococcus* (genus) and *Eggerthella* (genus) *Acetitomaculum* (genus), *Faecalicoccus* (genus), *Kopriimonas* (genus), Verrucomicrobia (phylum), *Flavonifractor* (genus), Christensenellaceae (family), Mogibacteriacea (family), *Akkermansia muciniphila*, Bacteroidetes, *Sutterella* (genus), *Butyricicoccus* (genus), *Lactobacillus*, *Bacteroides*, and *Alistipes*^36^

Polyphenol intake was associated with beneficial bacteria including *Ruminococcus bromii* assisting in resistant starch degradation in the human gut.^36^ *F. prausnitzii*, *Roseburia* and *Anaerostipes* produce butyrate, which stimulates production of mucins that improve gut integrity.^36^ Both *Eggerthella lenta* and *Bacteroides uniformis* can break down resveratrol into dihydroresveratrol, Proteobacteria including Gammaproteobacteria (class) metabolizes uric acid, and *Akkermansia* has been consistently associated with reduced fat mass.^36^ Some polyphenols can reduce the detrimental effects of potentially harmful microbial by-products. For example, the glycoside Allicin inhibits gut microbial production of pro-inflammatory TMAO^5^

In parallel to these prebiotic and health-promoting characteristics, *Lactobacillus*, *Bifidobacteria* and other commensals also halt the growth of, or outcompete pathogenic microbes.^36^ Clostridia (class) and some *Clostridium* spp. were reported as being substantially inhibited by polyphenols in one systematic review^14^ and in human and preclinical trials (Supplementary Table S6). This association was attributed to procyanidins and the catechins in foods like green and black tea.^36^ Other pathogens reported to be inhibited by phytochemicals included *Salmonella* (genus) and *Bacteroides*.^14^ The different types of polyphenols exert their respective effects by preferentially binding to specific bacterial cell membrane types, altering functional aspects of the membrane thereby preventing bacterial growth. Other mechanisms of action include changing the pH or releasing signal molecules that are not conducive to the growth of pathogenic bacteria.^161^ Some included studies reported no statistically significant associations between phytochemicals and microbiota but this could generally be explained by the studies being either under-powered, short-term studies without genus-level analysis or in ‘healthy’ populations, without existing dysbiosis.^36^

Naturally occurring food chemicals implicated in food intolerances include salicylates, amines, and glutamates. Although interaction with chemoreceptors is the most commonly proposed mechanism of action for natural food chemicals in gastrointestinal disorder management,^162^ salicylates, biogenic amines and glutamates (see also protein section) are each reported to have associations with gut microbiota^132^ and microbial metabolites.^30,133^ Further research is warranted to determine whether natural food chemicals act directly on chemoreceptors or whether microbiota or bioactive microbial metabolites play any role in etiology or symptom induction in these disorders.

### Food additives and the microbiota

Two reviews^38,39^ and 43 individual studies^105–134,152,153^ reported an association between microbiota and an emulsifier, thickener, stabilizer, preservative, flavoring, sweetener or food coloring (Supplementary Table S7). A high proportion of studies investigating the effects of food additives on the gut microbiota have been animal^38,118^ or in vitro studies,^106,130^ with human studies conducted to date being disease-specific.^163^ The emulsifiers carboxymethylcellulose and polysorbate-80 induced dysbiosis in one review that incorporated studies using animal and human models,^38^ with inconsistent findings reported in primary studies with human subjects or samples.^105,106^

The only other association consistently reported at the systematic review level was for maltodextrin, which was associated with altered microbiota Firmicutes, Bacteroidetes, *Lactobacillus* or *Bifidobacterium* in half of the 42 studies in one review.^39^ Maltodextrin is a food thickener and stabilizer that is often used as placebo for the control group in microbiome interventions, so it is notable that research indicates that this additive is not inert and independently influences the microbiota.^112,116^ It was notable that a daily dose of 2 g of the flavor enhancer monosodium glutamate resulted in no significant microbiota effects in one study.^152^

Overall, beneficial *Clostridium leptum* and *Blautia coccoides* seemed particularly susceptible to food additives, with reduction in response to emulsifiers, colorings, detergent and preservatives.^106^ The addition of dishwashing detergent to human fecal samples negatively affected many bacterial types, resulting in increased pathogenic *E. coli*, *Shigella* and *Klebsiella*, increased *Bacteroides*/*Prevotella* ratio,^106^ decreased relative abundance and decreased beneficial butyrate-producing *Bifidobacterium*, Firmicutes, *B. coccoides* and *C. leptum*.^106^ Carrageenan emulsifier and cinnamaldehyde flavoring exerted similarly detrimental effects on microbiota as detergent, although fewer microbial species were reported to be affected.^106^ Thickening agents gum Arabic^107,108^ and xanthan gum^109^ were reported to be cleaved by specific primary degrading bacteria, producing metabolites for secondary degradation by other bacteria. These gums therefore exert differential effects on microbiota depending on the relative abundance of primary and secondary degraders. In two studies that tested the effects of preservatives on human fecal samples, pathogenic bacteria were generally increased, with detrimental effects on commensals.^106,110^ The natural preservative nisin exerted a beneficial effect by inhibiting *Clostridium difficile* and altering gram-positive bacteria^111^ (Supplementary Table S7).

Sweeteners that have been assessed for association with gut microbiota include aspartame (amino acid derived), trehalose (non-reducing disaccharide), stevioside (steviol glycosides), erythritol, xylitol, maltitol, lactitol (sugar alcohols), saccharin (chemical compound) sucralose (chlorinated sucrose) and polydextrose (glucose polymer, contains sorbitol). Study design varied considerably in terms of the dosage of sweeteners, method of analysis for associations and testing of single^106,116,117,128^ or combinations of sweeteners.^106,118^ Of the sweeteners identified as being associated with gut microbiota in this review, those reported to have a detrimental effect on microbiota included trehalose which increased *C. difficile*,^116^ and sucralose,^119^ saccharine^119^ and a combination of saccharine, sucralose and aspartame that had a dysbiotic effect on microbiota.^106^ Stevioside, sugar alcohols, aspartame and acesulfame-K^112^ were reported to influence gut bacteria^112^ and bacterial metabolites.^106^ The polyol sweetener mannitol was also reported to alter the metaproteome across microbiomes, whereas erythritol did not significantly alter metaproteomes in one study.^153^

Evidence relating to the influence of food coloring intake on gut microbiota is restricted to animal studies and in vitro studies using human fecal samples. Comparisons between studies are limited by the diverse study designs with high variability between colors investigated, dosage and sample preparation. The influence of colorants on microbiota included altered growth of *Bacteroides*, *Bifidobacterium*, *Clostridium*, *Enterococcus*, *Escherichia*, *Lactobacillus* and *Ruminococcus*^131^ and *Saccharomyces*^129^ species, decreased microbial metabolism of isobutyrate, isovalerate and methanol,^130^ decreased production of acetate, butyrate and propionate^130^ and increased bacterial degradation liberating potentially carcinogenic metabolites.^164^

### Individual foods and the microbiota

Foods and beverages assessed for associations with microbiota separately from their nutrient components were predominantly investigated for a prebiotic or probiotic effect. Items included nuts, specific nut varieties, alcoholic drinks, coffee, and fermented foods or drinks, and were reported across three reviews^14,47,48^ and fourteen primary studies^54,141–144,146,147,149–151,158,159,165,166^ (Supplementary Table S8)

The combined effect of the fiber, phytochemical and fatty acid content on microbiota was assessed for individual nut varieties in some studies^48,141,143,144,146,147,149,165^ and collectively in two reviews.^47,48^ The influence of these foods on microbiota was generally prebiotic and consistent with reported separate effects of constituent fibers, phytochemicals, and fatty acids. One review reported that significant increases in *Roseburia* and *Clostridium* were negated if walnut studies were removed from analyses.^47^

One review^14^ and one primary study^151^ reported on the influence of alcoholic drinks on microbiota, with the findings generally consistent with those reported for phytochemicals (see Section 6). In one study, red wine consumption increased Proteobacteria, Firmicutes and Bacteroidetes regardless of whether it contained alcohol or not, but dealcoholized red wine also increased Fusobacteria (phylum).^151^

Analysis of stool samples from 6,811 individuals from the American Gut Project revealed statistically significant differences in beta diversity and differential taxa between fermented food consumers and non-consumers, with the metabolome of fermented food consumers enriched with putatively health-promoting conjugated linoleic acid.^158^ Two similar fermented dairy products were reported to influence microbiota.^54,142^ Buttermilk increased bacterial diversity^142^ and yogurt increased *Lactobacillus*, *Bifidobacterium* and *Streptococcus thermophilus*.^54^ A diet rich in fermented foods steadily increased microbiota diversity and decreased inflammatory markers in another intervention study^159^

### Dietary factor classification by dietary assessment and microbial degradation

Dietary factors in this review identified as being associated with microbiota were categorized as macronutrients (carbohydrate, fat, protein), micronutrients, other bioactive food compounds, food additives and specific foods. The foods or nutrients that were assessed for associations with microbiota in the current review could therefore be classified into four broad groups based on human digestibility, microbiota degradability and whether they can be assessed in the human diet using existing methods:

1. Routinely analyzed using standard food composition database and relevant to microbiota because they are not absorbed for human metabolism during digestion. These were classified as ‘degradable, assessable’ and included carbohydrate, fat and protein;
2. able to be analyzed using standard methods but predominantly absorbed in the small intestine so may be of less or indirect relevance to large bowel microbiota. These were classified as ‘digestible, assessable’ and included carbohydrate, protein, fat and micronutrients;
3. foods or nutrients regularly investigated in relation to gastrointestinal microbiota but that can only be assessed and analyzed in isolation, not as part of the whole food matrix or whole-of-diet analysis, because they are not listed in food composition databases. These were classified as ‘degradable, individually assessable’ and included proteins and protein components (peptides and amino acids), or:
4. foods, food components or nutrients that are important or potentially relevant to gastrointestinal microbiota research but were rarely assessed or only reported in animal studies. These were classified as ‘degradable, not assessed or not assessable’ and included food additives, bioactive compounds and specific foods.

## Discussion

This systematic review synthesizes existing research findings regarding relationships between dietary intake and the microbiota, and progresses understanding of which food components may be most relevant to diet-microbiota research. It is evident from this review that some food components that are not quantified in current food composition databases do impact microbiota composition, function and potential health influences. Studies investigating such microbiota-relevant food components therefore currently need to analyze food components of interest individually, which is expensive and inefficient and has the potential for error. This review highlights the need for more comprehensive methods to assess and analyze intake of specific food components relevant to microbial metabolism. This level of detail in dietary assessment for diet-microbiota research will be necessary before it is even possible to consider future goals of personally tailored microbiota-specific nutrition therapy for maintaining or improving health status.

### Degradable, assessable nutrients (carbohydrate, protein, fat)

Some food components routinely analyzed using standard dietary assessment methods are highly relevant to microbiota because they are not absorbed during human digestion and metabolism. The most obvious example is the expected and evident positive influence of a high or increased dietary fiber intake on bacterial profile, microbiota diversity and metabolite production.^14,25,74–77^ This prebiotic effect of fiber is consistent with existing literature and consensus in the field.^3,50^ The strong alignment of microbiota-relevant dietary factors that are comprehensively captured in existing dietary assessment and analysis methods was evidenced by the high proportion of studies that reported on these dietary factors and high number of reported fiber-microbiota associations.

As dietary fibers are commonly found in high carbohydrate foods, it can be difficult to conclude whether observed effects are related to total dietary fiber intake or the whole food containing the carbohydrate or other nutrients associated with those foods. Total dietary fiber was the most commonly reported fiber measure in studies included in this review, but various types of dietary fiber may differentially impact the microbiome, as does the form in which they are consumed (i.e., supplemented or within a whole-food matrix). This highlights the need for further characterization of the physicochemical characteristics and combinations of fibers, doses and duration of intake to achieve clinically meaningful GI health benefits.^167^

The overlap between effects of prebiotic fibers and high FODMAP diets and opposing findings with low FODMAP diets on microbiota are expected, given the prebiotic capabilities of oligosaccharide FODMAPs.^168^ Many bacteria can break down short-chain oligofructose but only specific *Bifidobacterium* spp. digest long-chain fructans.^168^ The individual effects of other constituent FODMAPs on human microbiota have not been as thoroughly investigated. The availability of fructose for bacterial degradation is highly determined by dietary load and subsequent availability of the fructose supply to bacteria, as well as the gut microenvironment, with availability of fructose being much higher in the duodenum compared to the large intestine.^169^ Lactose degradation is determined by relative availability of lactase enzyme within the villi in the duodenum. Although lactose, fructose and polyols are grouped together as osmotic rather than gas producing FODMAPs, they each influence gut microbes differently, and individual polyols may also have different effects.

Other dietary associations with microbiota that were expected and readily analyzed included very high intakes of dietary fat^96,97^ or protein^14^ that could not feasibly be completely absorbed in the small intestine. Amino acids are consumed in food both as ‘free’ form or as part of peptide chains that are enzymatically digested to amino acids. Some microbes also cleave peptides to amino acids or ferment amino acids, producing microbial fermentation metabolites, including ammonia, amines, phenols, indoles, and hydrogen sulfides.^50^ The wide range of reported protein-microbiome associations is partially attributable to the diversity of protein and amino acid structures, and the relative ability of different microbes to cleave bonds between amino acids or to utilize their nitrogen or sulfur. Mechanistic, in vitro and some experimental studies have investigated microbial metabolism of amino acids^46,54,68,69^ and identify microbiota-mediated degradation pathways^51^ and dominant amino acids metabolisers in each part of the digestive tract.^46^ Very few studies have reported on associations between dietary amino acid intake and microbiota because the necessary analysis methods are not available.

The implications of increased availability of fats and proteins to microbiota in the large bowel as a result of dietary manipulation, nutrient-binding medications or cooking methods are interesting to consider and plausible to analyze. Although measuring the exact amount of specific nutrients available for microbial degradation is not yet possible, the effect of very high intake of particular macronutrients can be assessed in interventions or in research with population subsets that follow specific dietary regimes that involve extreme macronutrient alternations, such as ketogenic diets.^170^ The effects of nutrient-binding medications are harder to assess because of the potential confounding effect of concurrently prescribed diet and lifestyle changes, and the possibility that medications may independently influence gut microbiota.^171^

### Digestible, assessable nutrients (carbohydrate, protein, fat, micronutrients)

Some nutrients or food components that can be assessed and analyzed using standard methods are relevant to upper but less relevant to lower GI microbiota because they are readily absorbed in the small intestine. Nutrients in this group from this review included digestible carbohydrate (total sugars, starches), total protein, some amino acids (e.g., tryptophan), dietary fat and most micronutrients. Far fewer associations were reported for total dietary proteins,^31,61^ carbohydrates^3^ and fats^61^ than for their constituents or foods containing these macronutrients. The relatively low number of diet-microbiota associations reported in reviews and primary studies for these nutrients is indicative of misalignment in timing between study design regarding microbiota sampling and dietary assessment or analysis methods.

**Figure 1.**
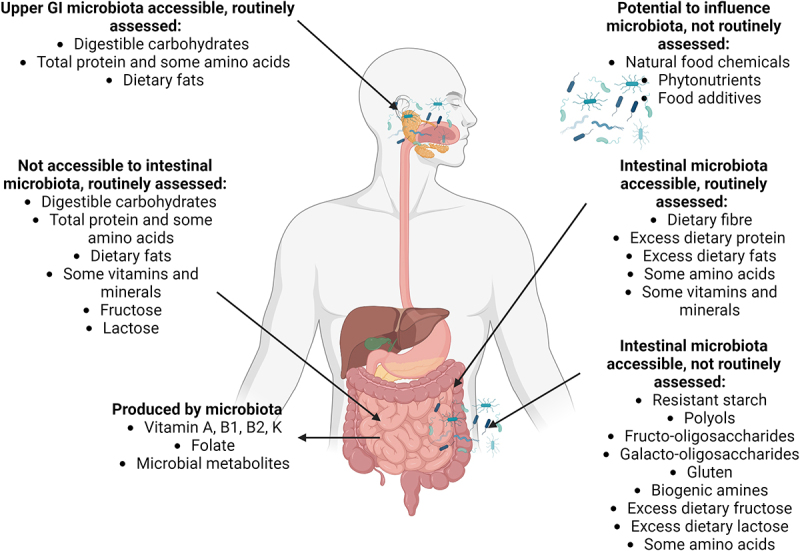
Accessibility of nutrients and other food components to intestinal microbiota at different sites within the human digestive tract, and associated dietary assessment methods.

As humans efficiently digest and absorb protein, the vast majority of dietary protein in an average diet is enzymatically digested and absorbed before the end of the small intestine, leaving little available for colonic microbial fermentation^172^ (Figure 1). However, dietary peptides can be a microbial fermentation substrate across the intestine from the oral cavity to the colon. *Bacteroides*, *Clostridium*, *Propionibacterium*, *Fusobacterium*, *Streptococcus*, *Bacillus*, *Lactobacillus* and Proteobacteria are all contain proteolytic bacteria involved in catabolism and recycling nitrogen compounds, including amino acids.^46,154^ Bacteria with proteolytic capability that can survive or proliferate in upper parts of the intestinal tract have a competitive advantage in terms of sourcing nutrients. For example, some oral microbes (*Rothia*, *Actinomyces odontolyticus*, *Streptococcus*) have gluten-cleaving capability,^155^ suggesting that bacterial cleavage of gluten may be initiated in the oral cavity before enzymatic digestion commences. Intestinal proteolytic digestion of gluten produces proline- and glutamine-rich polypeptides that can be cleaved by specific GI bacteria^32,156^ and *Pseudomonas* spp. including *P. aeruginosa* have gluten-degrading capabilities.^64,157^

Created in BioRender

Micronutrients contribute to human and gut microbial health. Assessing associations between micronutrient intake and microbiota is complex because of the bi-directional nature of influence between bacteria and some vitamins or minerals as some microbiota utilize micronutrients remaining from human digestion and some contribute to production of essential vitamins.^14^ For example, the function of certain gut microbes is dependent on vitamin B availability^14,173^ and the relative abundance of some microbes is associated with vitamin E intake.^174^ Alterations to gut microbiota have been reported following supplementation with vitamins B2 and B3,^174^ vitamin B12^33^ vitamin D,^34,35^ iron,^175^ calcium and phosphorous.^174^ Vitamins reported to be produced by gut microbes included vitamin A,^14,176^ vitamins B1 and B2,^177^ folate^14^ and vitamin K.^14^ Microbe composition and activity may also influence mineral absorption. Bioavailability of iron,^175^ calcium and phosphorous^174^ have been reported to improve as a result of SCFAs reducing the pH in colon. Beneficial *Lactobacillus* spp. increase bioavailability of magnesium, which in turn improves the composition of gut microbiota.^174^ Despite awareness of these bidirectional relationships, micronutrient bioavailability to microbiota and human hosts are not as well characterized.^173^

Although intake of micronutrients from food and supplements can be measured using standard dietary assessment methods, this review highlighted the considerable potential for improved characterization of the relationships between micronutrient bioavailability and microbiota in human hosts.^173^ For example, body stores and pools of some micronutrients are higher than indicated by dietary intake, suggesting they are produced in the colon by microbiota then absorbed.^173^ Genetic abnormalities, such as vitamin D receptor deficiency, have also been used to investigate gut dysbiosis. Bioavailability and modulation of micronutrient status via microbiota are emerging and important areas of research. The implications of improved understanding of these relationships are broad, extending across dietary intake, nutritional supplements, pharmaceuticals and disease management. The lower proportion of studies that reported no significant microbiota alterations for the specified nutrients is consistent with conclusions of Jardon et al. (2022), who indicate a need for improved understanding of the intersect between human digestion and microbial fermentation in nutrient metabolism.^10^ They propose that assessment and analysis of GI site-specific fermentation will advance our ability to influence interplay between diet, gut microbiota and metabolic processes.^10^ It is likely that the lack of specificity when measuring total macronutrients, rather than component saccharides, amino acids and fatty acids, results in lack of sensitivity to changes in gut microbiota. Such changes result from delivery of specific nutrients to symbionts that possess specific systems for their metabolism.^178^

### Degradable, individually assessed nutrients (protein, including peptides and amino acids)

The third group of food components identified in our review were those predicted to influence the GI microbiota but were usually assessed and analyzed in isolation, rather than as part of the whole food matrix. This limits attribution of causation to the individual food or nutrient.

Gluten is an example of a food component that is highly relevant to GI health and the microbiota, but unable to be analyzed using current national food composition databases. Analyzing the presence or absence of gluten using ELISA^179^ is not useful for determining total dietary gluten intake, but is the only widely available method used in the food industry to date. Investigating the influence of gluten on microbiota is further complicated because much of the research is related to avoidance of gluten by people with celiac disease, or for other health-related reasons. This could mean that metabolic factors influence intestinal microbiota responses to gluten, in addition to the changes induced by altered dietary gluten intake.^62^

Amino acid-based natural food chemicals (glutamates, biogenic amines) and microbial fermentation metabolites (short- or branched chain fatty acids, phenols, amines, sulfides, ammonia and trimethylamine N-oxide) need to be considered in diet-microbiome analysis because of their potential implications for the gut-microbiome-brain axis and related metabolic and health outcomes.^14,40,73^ Histamine is of particular relevance in relation to GI conditions, being implicated in inflammatory mast cell activation^180^ and chemosensor activation.^181^ The influences of potentially immunogenic peptides resulting from microbial cleavage of dietary proteins may also have a role in immune-related GI manifestations.^182^ Differentiating the relative influence of dietary histamine in the context of diet-microbiota associations is complicated by the fact that mast cells and many bacteria, as well as some yeasts and molds have the capacity for histamine formation.^41^

### Other foods, food components and nutrients with potential to influence microbiota (food additives, bioactive compounds, specific foods)

A fourth group of food components important to GI microbiota research were identified, which were rarely quantified or only reported in animal studies. These food components included food additives and microbiota-relevant bioactive food compounds such as phytochemicals, food additives, salicylates, and glutamates.

Incorporating all chemicals in plants, phytochemicals have varying, but potentially profound influences on gut microbes that are exerted by inhibiting growth of pathogenic microbes, producing bioactive microbial metabolites and promoting growth of commensal microbes.^36^ Phytochemicals can be grouped by their structure, function or by their color^36^ and their health-promoting efficacy depends on bioavailability, structural complexity, and positioning within the food matrix. To optimally assess the influence of phytochemicals in the context of the food matrix, high phytochemical foods need to be consumed as part of a whole diet, then coded and analyzed within the same food composition database as other nutrients being reported.

The salicylate and glutamate contents of foods have been semi-quantified on low food chemical dietary guidance, but not in the context of diet-microbiota assessment. To determine whether associations between natural food chemicals and microbiota exist, these bioactive compounds need to be assessable in the context of the whole diet.

Data sources used to quantify dietary intake of food additives vary depending on the additive or specific health condition being assessed, and range from population-level data linked to branded food databases to individual-level assessment using food frequency questionnaires, 24-h recall or food diaries.^183^ Food additive analysis methods need to become more sensitive and specific for accurate assessment of the effects of food additives on the gut microbiota.

It was evident from this review that some dietary factors influence the microbiota via less direct mechanisms. For example, associations between fat and microbiota are likely to be attributable to increased bile acids in response to dietary fat ingestion, particularly saturated fat. Although nearly all fatty acids are absorbed in the small intestine, bile released in response to dietary fat intake can impact on microbiota throughout the digestive tract. Medications that bind fat can also alter microbial fermentation potential because they are not absorbed in the small intestine and become a fermentation substrate in the colon. It remains a substantial challenge in this field to differentiate the direct effects of macronutrient intake on microbiota from the indirect effects of metabolic factors, disease states or predisposition and medications.^184^

The relationships between fermented foods and microbiota are complex, with the findings that diet steadily increased microbiota diversity consistent with a narrative review that reported high fermented food intake to influence more than 50 bacterial species across 20 studies.^185^

### Microbiota sampling

Although microbiota sampling and microbiome analysis methodologies were not the primary focus of this review, some findings did highlight potential future directions that were particularly relevant to assessment of diet-microbiota associations. For example, reported diet-microbiota associations needed to be considered in relation to the location within the digestive tract being investigated. Intestinal microbes that differentially populate the various niche environments of the digestive tract have different fermentation capabilities and substrate preferences, which has implications for analysis and interpretation of diet-microbiota associations.^178^ Few studies in this review reported on upper GI microbiota data, which would be more specific and sensitive for analyzing microbiota associations with some nutrients.

Niche environments also exist in between the mucosal surface and lumen in the different compartments along the GI tract. In the current review, the vast majority of microbiota and microbiota data originated from stool samples and are therefore representative of luminal colonic microbes, which may be considerably different to the diversity and abundance of microbial species in the colonic mucosa or the lumen or mucosa of other parts of the digestive tract.

Another consideration is matching the timing of stool sampling to dietary intake. Microbial species respond at different rates to dietary changes, so alignment of sample collection and dietary assessment is important. In their longitudinal study with 34 individuals who provided a stool sample daily for 17 days, Johnson et al. (2019) reported that microbiota composition aligned best with multiple days of dietary data history, aligned more strongly with food than nutrients and that microbCreated in BioRenderial responses to diet were highly individual.^148^ Timing of stool sample collection is not often outlined in study methods, which may influence accuracy of reported outcomes.

### Improving dietary assessment and analysis in microbiome research

There is considerable scope to improve dietary assessment methods in diet-microbiome research to differentially measure associations with mucosal or luminal microbiota from samples collected throughout the digestive tract from mouth to anus. Accurate, longitudinal dietary intake assessment and analysis, combined with collection of multiple microbiota samples from relevant compartments in the digestive tract will contribute to more reliable, rigorous diet-microbiome data. Baseline gut microbial characteristics and habitual dietary intake may predict an individual’s response to dietary interventions. It is therefore crucial that both dietary data and microbiota samples are collected at this timepoint, as well as during and post-study periods, in order to better understand factors influencing intervention responses.^186^ Analysis methods may also substantially influence reported associations, depending on whether individual or group-level analysis has been used. For example, Tap et al. (2015) reported no significant inter-individual differences but significant intra-individual genus-level microbiota differences in a study investigating differences in microbiota with low or high fiber intake.^77^ Understanding the mechanisms of the differential responses to diet is essential to move forward in the field of precision nutrition.^10^

Some interesting considerations evidenced from this review include the need for more research in which ‘target’ foods are consumed and analyzed within the whole diet context, the exciting potential for progress in this field and the substantial opportunity for secondary analysis of existing dietary data collected in diet-microbiome research.

Two substantial shifts in dietary assessment methodology are needed for diet-microbiome research to progress. The main shift from a dietary assessment perspective is to consider what is not digested by human digestive enzymes, and what might be degraded in different parts of the digestive tract to where enzymatic digestion occurs. For example, gluten degrading microbes are present in the oral cavity, so it is feasible that gliadin peptides from gluten degradation are present in the esophagus.^187^ The change in focus from a dietary analysis viewpoint is to consider what data from whole-diet intake assessment can and cannot be quantified using available dietary analysis methods, and how data from the wide range of different sources described in this review could potentially be consolidated. These major considerations will require combined dietary assessment, microbiome and metabolomic expertise and considerable investment, but could stand to contribute to quantum diet-microbiome knowledge gains, applications and opportunities.

### Recommendations

1. *To optimize analysis of diet-microbiome associations*: Highly specific microbiota data needs to be compared with equally robust and specific dietary intake data. Where dichotomous dietary categorization is appropriate to the research question or necessary due to budget constraints, it’s use in microbiome research could be enhanced by use of standardization of macronutrient proportions and energy intake categorization and by use of validated, accessible tools for delineating dietary patterns. Improved use of multiomics modeling of metabolic pathways specific to individual microbiome signatures could facilitate precision nutrition to modulate metabolites associated with human health.^171^
2. *Optimizing diet assessment and analysis in study design*: In future studies that involve shotgun metagenomics, it is recommended that an expert in dietary assessment is involved in planning the dietary assessment and analysis to ensure the research questions can be answered using the methods employed and that use of the dietary data is optimized in the context of study variables. Additionally, future studies should ensure that dietary assessment methods report details of the food composition database used to analyze intake. Consensus methods and/or a core outcome set for diet–microbiome studies would facilitate the achievement of this recommendation.
3. *Opportunities for secondary analysis*: A high proportion of included studies reported dietary data collection that far exceeded what was used in diet-microbiota analysis and reported as study findings. This suggested there is a considerable dietary data across studies already conducted that could be used for secondary analyses. We recommend that researchers consider involving dietary assessment specialists to optimize dietary data use, and potentially consider retrospective, secondary analyses. For example, it is possible for data from food frequency questionnaires to be disaggregated, reanalyzed using additional microbiota-relevant dietary data and then for associations with microbiota or microbiome data to be reevaluated.
4. *Matching dietary assessment methods to microbiome sampling methods*: Accurate dietary intake quantification will reduce systematic and random errors. The choice of dietary assessment method needs to be appropriately aligned with research question in relation to microbiome sampling and analysis. For example, food frequency questionnaires are appropriate for assessing associations between dietary patterns and the microbiome, whereas repeat 24-h recalls are more appropriate to assess short-term changes in microbiome in relation to daily variability in dietary intake.
5. *To improve dietary assessment of fat-microbiota associations*: Consensus around grouping of fatty acids for analysis is needed. Approximation of usual dietary intake requires consumption of fat within the food matrix, rather than analysis of supplementary fats only. Particular consideration should be given to the production and influence of bile and dietary fibers is recommended.
6. *Improved assessment and analysis of carbohydrate-microbiota associations*: This will depend on increased consideration of bacterial metabolism or foods in addition to usual consideration of enzymatic digestion, and consolidation of data from the range of industry and research sources from which it is currently drawn. Considerable progress in understanding of carbohydrate-microbiota associations could be made by conducting secondary analysis of already collected data, but using a consolidated analysis database that includes whole grain, resistant starches, fiber and FODMAP data.
7. *To improve dietary assessment of protein-microbiota associations*: More accurate tools for assessing dietary intake of amino acids and microbiota-relevant peptides such as gluten and gliadin are needed.
8. *Combining comparator data to increase accuracy*: Use of dietary intake in combinations with phenotypic and genotypic profiles, and with microbial metabolites and metabolomic data will increase accuracy for assessment of associations with microbiota data.
9. *Differentiating animal and human studies*: Animal and human studies are not clearly defined or differentiated in the findings of all trials or reviews, adding further complexity to analysis of associations. It was also evident that animal trials are used in cases where the safety of a new food additive or supplement has not been established. For example, one animal study reported that high doses of inulin induced microbiota-related hepatic damage, and cautioned high-dose supplementation in humans.^84^ These findings are inconsistent with the findings from human studies on inulin supplementation that used physiologically justifiable doses of inulin, but nevertheless warrant further investigation.
10. *Differentiating digestive health, disorder, or disease state of participants*: Although the review focused on healthy individuals, some studies included or compared healthy individuals and those with gut disorders. It was interesting that two reviews reported similar effects of prebiotic fibers on bacterial profiles between healthy individuals and those with intestinal diseases.^16,168^

### Strengths and limitations

This systematic review comprehensively assesses associations between diet and microbiota in human studies, describing relevant dietary assessment methods where possible. As the aim of this review was predominantly focused on capturing all reported diet-microbiota associations, systematic reviews and primary studies were included, so additional screening was conducted to mitigate overlap between reporting of review and primary study findings. It was not possible to consistently report whether 16S or MGS was used for microbial sequencing, as it was not always reported. While this is a limitation in terms of comparability between studies, the intention of this review was to describe the range of food constituents that have been assessed and those that should be considered in the future for more sensitive and specific analysis of diet-microbiota associations.

## Conclusion

This systematic review highlights considerable opportunities to improve diet-microbiome research methodology. Dietary assessment methodology needs to expand to include microbiota-relevant data and integrate with nutritional metabolomic data, which requires dietary assessment expertise. Study designs need to align dietary assessment methods with standardized microbiota sample collection and analysis, using deeply phenotyped participant cohorts. Refinement and consolidation of food composition databases should include microbiome-relevant data, particularly from human intervention studies. A substantial shift in dietary and nutritional metabolomic quantification methodology has the potential to make microbiome-specific nutrition therapy a reality.

## Materials and methods

This review involved a systematic literature search and data extraction process, with narrative synthesis of extracted data.

### Step 1: systematic search to identify studies for inclusion

PUBMED search: ((((((((((((((((fiber) OR (fiber)) OR (carb*)) OR (FODMAP)) OR (protein)) OR (fat)) OR (food additive)) OR (emulsifier)) OR (food chemical)) OR (diet[MeSH Terms])) OR (nutritive value[MeSH Terms])) OR (additive, food[MeSH Terms])) AND (microbiota)) OR (microbial)) OR (microbiome)) AND (gut)) OR (gastrointestinal microbiome[MeSH Terms]).

Systematic reviews that addressed at least one dietary factor in relation to the human gut microbiome were included. While focused on healthy individuals, reviews were included if they included both human and animal data, or they compared healthy individuals and those with gastrointestinal conditions, if these data could be differentiated and extracted. The search state date of January 1, 2010, was based on the rationale that this would result in a higher proportion of studies that reported MGS and less 16s only microbiome data, and therefore be more comparable. Reviews were excluded if they focused on very specific population groups (e.g. people who had undergone bariatric surgery, cancer treatment, dialysis).

Two reviewers (JH, KD) independently screened titles and abstracts for relevant papers, with conflicts discussed to achieve consensus on inclusion and exclusion of retrieved titles.

### Step 2: identification of primary studies for inclusion

1. Data from primary studies identified in the search were extracted to data extraction tables
2. Studies identified in systematic reviews retrieved above, where only one study was identified as reporting on a diet-microbiome association of interest, were extracted as primary studies.
3. The reference lists of narrative reviews identified during literature searches, including those identified via Step 1, were screened by one reviewer (JH) for reporting of other diet-microbiome factors. Any new studies identified were extracted as primary studies.
4. Dietary factors with potential microbiome associations (identified in preclinical studies) were identified, with individual searches for relevant human studies conducted in PubMed using a similar search strategy as described above, without AND (systematic review).

### Step 3: data extraction and synthesis

Data were extracted by one reviewer (JH) to an excel spreadsheet with one main spreadsheet, then discussed and grouped (JH, KD) before individual sheets (KD) were created for each dietary factor identified (dietary profile, carbohydrates and fibers, proteins, fats, bioactive food compounds, food additives and individual foods). Data retrieved from systematic reviews were differentiated from primary studies, with the number of studies from the review that reported on each specific dietary factor reported as identified as (SR, x or x/y studies) depending on whether or not the number of studies that reported non-significant findings were specified. Study design and characteristics relating to dietary assessment and analysis methods were also extracted, including retrieval of information from protocols and supplementary materials, if indicated in the article (Supplementary Tables S1–S8). Extracted data were checked by a second researcher to ensure that findings from systematic reviews and primary studies that may have contributed to systematic reviews were differentiated as far as possible, to avoid replication of reported findings. Data from reviews and primary studies were then consolidated, with the number of studies reporting on each dietary factor and number of reported associations with bacterial numbers, diversity or abundance tallied and presented as tables and narrative reporting in the manuscript.

Consolidated data describing diet-microbiome associations and dietary assessment methods were then described narratively. Supplementary tables that include diet-microbiota associations were prepared and are available on request by e-mail to the corresponding author.

## Supplementary Material

Supplemental Material

## Data Availability

The data that support the findings will be available in Open Science Foundation at https://osf.io/mw2ek/following a 12-month embargo from the date of publication to allow for commercialization of research findings.
