# Supplementary material for: Diet-microbiota associations in gastrointestinal research: a systematic review
Source: Gut Microbes. 2024 May 9;16(1):2350785. doi: 10.1080/19490976.2024.2350785 (PMC11093048; doi:10.1080/19490976.2024.2350785)
Supplement: Supplemental Material [file KGMI_A_2350785_SM1002.zip › Supplementary tables diet microbiome review_FINAL.docx]

**Supplementary Table 1: Associations between dietary patterns and microbiota reported in reviews and primary studies**

| **Dietary profile** | **Review or primary study** | **Number of studies (SR) or participants (PS) and study design** | **Associations (all taxonomic levels)** | **No. studies in review reporting differences** |
| --- | --- | --- | --- | --- |
| Plant-based diet | SR [22] | 9 studies | 2 | 2 |
| Plant-based diet | SR [23] | 16 studies | 0 | 0 |
| Plant-based diet | SR [24] | 34 studies | 0 | 0 |
| Plant-based diet | SR [13] | 16 studies | 16 | 11 |
| Plant-based diet | PS [54] | 1098; cross sectional | 6 | N/A |

| Plant-based diet | PS [56] | 29; cross sectional | 4 | N/A |
| --- | --- | --- | --- | --- |

| Plant-based diet | PS [57] | Lab, bacteria, genes | 5 | N/A |
| --- | --- | --- | --- | --- |
| Plant-based diet | PS [137] | 10: human intervention | 6 | N/A |
| Western diet | SR [14] | 86 | 2 | 6 |
| Western diet | PS [54] | 1098; cross sectional | 5 | N/A |
| Western diet | PS [58] | 11; human intervention | 9 | N/A |
| Western diet | PS [59] | 19; human intervention | 5 | N/A |
| Western diet | PS [60] | 14; human intervention | 4 | N/A |
| Western diet | PS [55] | 22; human intervention | 5 | N/A |
| Western diet | PS [53] | 27; human faecal sample | 5 | N/A |
| Low calorie diet | SR [43] | 7 (130 participants total) | 8 | 7 |
| Low calorie diet | SR [14] | 86 | 3 | 1 |
| Low calorie diet | SR [24] | 34 | 2 | 1 |
| Low calorie diet | PS [60] | 14; human intervention | 8 | N/A |
| Low calorie diet | PS [61] | 14; human intervention | 2 | N/A |

N/A: Not applicable, No.: number, PS: Primary study, SR: Systematic review

**Supplementary Table 2: Associations between dietary carbohydrates (including fibres) and microbiota reported in reviews and primary studies**

| **Dietary factor** | **Review or primary study** | **Number of studies (SR) or participants (PS) and study design** | | **Associations (all taxonomic levels)** | | **No. studies in review reporting differences** | |
| --- | --- | --- | --- | --- | --- | --- | --- |
| Fibre | SR [14] | 86 studies | | 5 | | 8 | |
| Fibre | SR [25] | 26 human studies | | 52 | | 6 | |
| Fibre | PS [74] | 9; human cross sectional | | 1 | | N/A | |
| Fibre | PS [76] | 2 subjects; observational | | 1 | | N/A | |
| Fibre | PS [77] | 19; human intervention | | 1 | | N/A | |
| Carbohydrate intake | PS [75] | 32; human cross sectional | | 4 | | N/A | |
| Carbohydrate intake | PS [58] | 11; human intervention | | 2 | | N/A | |
| Carbohydrate intake | PS [59] | 19; human intervention | | 2 | | N/A | |
| Carbohydrate intake | PS [60] | 14; human intervention | | 2 | | N/A | |
| Resistant starch | SR [14] | 86 studies | | 3 | | 1 | |
| Resistant starch | SR [17] | 46 studies | | 1 | | 2 | |
| Resistant starch | PS [61] | 14 human intervention | | 2 | | N/A | |
| Resistant starch | PS [60] | 14 human intervention | | 1 | | N/A | |
| Resistant starch | PS [78] | 10 human intervention | | 6 | | 3 | |
| Prebiotic fibres | SR [26] | 29 studies | | 2 | | 15 | |
| Prebiotic fibres | SR [27] | | 29 studies (1444 participants) | | 1 | | 11 |
| Prebiotic fibres | SR [17] | 46 studies | | 7 | | 13 | |
| Prebiotic fibres | SR [14] | 86 studies | | 5 | | 8 | |
| Prebiotic fibres | SR [42] | N/A | | 4 | | N/A | |
| Prebiotic fibres | PS [80] | 34; human intervention | | 1 | | N/A | |
| Prebiotic fibres | PS [81] | 18; human faecal intervention | | 1 | | N/A | |
| Prebiotic fibres | PS [82] | 10; human XC | | 2 | | N/A | |
| Prebiotic fibres | PS [83] | 49; human intervention | | 1 | | N/A | |

| Prebiotic fibres | PS [84] | Preclinical | N/A | N/A |
| --- | --- | --- | --- | --- |

| Prebiotic fibres | PS [136] | 34; human intervention | 5 | N/A |
| --- | --- | --- | --- | --- |
| Prebiotic fibres | PS [140] | 21: human intervention | 2 | N/A |
| Wholegrains | SR [44] | 16 studies | 5 | 6 |
| Wholegrains | SR [29] | 84 studies | 2 | 7 |
| Wholegrains | SR [28] | 35 studies (n=1080) | 2 | 4 |
| Wholegrains | SR [17] | 46 studies | 1 | 14 |
| Wholegrains | PS [85] | 32; human intervention | 2 | 1 |
| Wholegrains | PS [87] | Lab: 3 human faecal samples | 1 | N/A |
| Wholegrains | PS [86] | 37; human intervention | 1 | 1 |
| Wholegrain fibres | SR [15] | 42 studies | 14 | 39 |
| Wholegrain fibres | SR [14] | 86 studies | 7 | 2 |
| Wholegrain fibres | PS [88] | 10; human intervention | 7 | N/A |
| Wholegrain fibres | PS [89] | 51; human intervention | 6 | N/A |
| Wholegrain fibres | PS [90] | 60; human intervention | 1 | N/A |
| Wholegrain fibres | PS [91] | 18; human intervention | 2 | N/A |
| FODMAP content | SR [16] | 15 studies | 5 | 6 |
| FODMAP content | PS [82] | 10; human cross-sectional | 2 | N/A |
| FODMAP content | PS [79] | 27; human intervention | 3 | N/A |
| Low FODMAP | SR [16] | 15 studies | 4 | 1 |
| Low FODMAP | SR [30] | 7 studies | 4 | 7 |
| Low FODMAP | SR [45] | 9 studies | 1 | 4 |
| Low FODMAP | PS [65] | 30; human intervention | 2 | N/A |
| Fructose | PS [92] | Lab, 3 human faecal samples | 3 | N/A |
| Fructose | PS [93] | Preclinical | Metabolites | N/A |
| Fructose | PS [94] | 52; human cross sectional | 2 | N/A |
| Lactose | PS [95] | 28; human (infant) intervention | 4 | N/A |
| Lactose | PS [82] | 10; human cross sectional | 3 | N/A |
| White bread vs rice | PS [135] | 7; human intervention | 2 | N/A |
| Advanced glycation end products | PS [150] | 65; human intervention | Metabolites | N/A |

N/A: Not applicable, No. number, PS: primary study, SR: systematic review

**Supplementary Table 3: Associations between dietary proteins and microbiota reported in reviews and primary studies**

| **Dietary factor** | **Review or primary study** | **Number of studies (SR) or participants (PS) and study design** | **Associations (all taxonomic levels)** | **No. studies in review reporting differences** |
| --- | --- | --- | --- | --- |
| Dietary protein | SR [14] | 86 | 5 | 3 |
| Dietary protein | SR [31] | 15 | 0 | 0 |
| Dietary protein | SR [160] | 85 | 3 | 2 |
| Dietary protein | SR [46] | 1; human faecal | 8 | 1 |
| Dietary protein | PS [154] | N/R | 8 | N/A |
| Dietary gluten | SR [32] | N/R | 5 | 3 |
| Dietary gluten | PS [155] | In vitro bacteria | 4 | N/A |
| Dietary gluten | PS [156] | 42; human cross sectional | 1 | N/A |
| Dietary gluten | PS [157] | 22; human faecal samples | 2 | N/A |
| Dietary gluten | PS [64] | 30; human cross sectional | 1 | N/A |
| Dietary gluten | PS [62] | 60; human intervention | 14 | N/A |
| Dietary gluten | PS [67] | 20; human intervention | 7 | N/A |
| Gluten free diet | PS [63] | 14; human intervention | 7 | N/A |
| Gluten free diet | PS [64] | 30; human cross sectional | 2 | N/A |
| Gluten free diet | PS [65] | 30; human intervention | 4 | N/A |
| Gluten free diet | PS [66] | 44; human cross sectional | 3 | N/A |
| Amino acids | SR [46] | 1; human faecal | 8 | 1 |
| Amino acids | SR [14] | 86 | 7 | 3 |
| Amino acids | PS [69] | N/R | 1 | N/A |
| Amino acids | PS [70] | 33; human intervention | 2 | N/A |
| Amino acids | PS [71] | 28; human intestine, cross sectional | 4 | N/A |
| Amino acids | PS [155] | In vitro | N/A | N/A |
| Amino acids | PS [139] | 64; cross sectional | 5 | N/A |

N/A: Not applicable, No. number, PS: Primary study, SR: Systematic review

**Supplementary Table 4: Associations between dietary fats and microbiota reported in reviews and primary studies**

| **Dietary factor** | **Review or primary study** | **Number of studies (SR) or participants (PS) and study design** | **Associations (all taxonomic levels)** | **No. studies in review reporting differences** |
| --- | --- | --- | --- | --- |
| Total and saturated fats | SR [14] | 86 | 8 | 4 |
| Unsaturated fats | SR [14] | 86 | 3 | 2 |
| Unsaturated fats | SR [18] | 5 | 2 | 2 |
| Dietary fat content | PS [96] | 98; human intervention | 1 | N/A |
| Dietary fat content | PS [97] | 88; human intervention | 3 | N/A |
| Dietary fat content | PS [98] | 217 human intervention | 2 | N/A |
| High saturated vs unsaturated fats | PS [99] | 109; human intervention | N/A (OTUs) | N/A |
| High saturated vs unsaturated fats | PS [160] | 531; human cross sectional | 4 | N/A |
| High saturated fat | PS [97] | 109; human intervention | 1 | N/A |
| Unsaturated fats | PS [100] | 126: human intervention | 1 | N/A |
| Unsaturated fats | PS [20] | 34: human intervention | 0 | N/A |
| Unsaturated fats | PS [74] | 9; human cross sectional | 1 | N/A |
| Unsaturated fats | PS [101] | 40; human cross sectional | 2 | N/A |
| Unsaturated fats | PS [103] | 94; human cross sectional | 2 | N/A |
| Unsaturated fats | PS [102] | 25; human intervention | 9 | N/A |
| MCT vs LCT | PS [148] | 34; human, 17 days of sampling | 0 (all NS) | N/A |

LCT: long chain triglyceride, MCT: medium chain triglyceride, N/A: Not applicable, No.: Number, NS: not significant, OTU: Operational taxonomic unit, PS: primary study, SR: systematic review

**Supplementary Table 5: Associations between micronutrients and microbiota reported in reviews and primary studies**

| **Dietary factor** | **Review or primary study** | **Number of studies (SR) or participants (PS) and study design** | **Associations (all taxonomic levels)** | **No. studies in review reporting differences** |
| --- | --- | --- | --- | --- |
| Vitamin A | SR [14] | 86 | b-carotene production | 2 |
| Vitamin B6 | SR [14] | 86 | 2 | 2 |
| Vitamin B9 (folate) | SR [14] | 86 | 1 | 4 |
| Vitamin B12 | SR [14] | 86 | 2 | 2 |
| Vitamin B12 | SR [33] | 22 | 4 | 11 |
| Vitamin D | SR [34] | 14; human | 6 | 14 |
| Vitamin D | SR [35] | 25 | 6 | 7 |
| Vitamin K | SR [14] | 86 | Produced by bacteria | 3 |
| Calcium and Phosphorous  Iron deficiency | PS [104] | 62; human intervention | 1 | N/A |

N/A: Not applicable, No. Number, PS: primary study, SR: systematic review

**Supplementary Table 6: Associations between bioactive compounds and microbiota reported in reviews and primary studies**

| **Dietary factor** | **Review or primary study** | **Number of studies (SR) or participants (PS) and study design** | **Associations (all taxonomic levels)** | **No. studies in review reporting differences** |
| --- | --- | --- | --- | --- |
| Phytochemicals | SR [27] | 29 studies (1444 participants) | 1 | 11 |
| Phytochemicals | SR [30] | 7 studies | 4 | 7 |
| Phytochemicals | SR [36] | 25 | 14 | 1 |
| Cocoa (flavanols) | SR [14] | 86 | 4 | 4 |
| Ellagitannins | SR [14] | 86 | 2 | 4 |
| Catechins | SR [14] | 86 | 5 | 3 |
| Catechins | SR [37] | 6 human, 15 animal | 1 | 1 |

N/A: Not applicable, No.: Number, PS: primary study, SR: systematic review

**Supplementary Table 7: Associations between food additives and microbiota reported in reviews and primary studies**

| **Dietary factor** | **Review or primary study** | **Number of studies (SR) or participants (PS) and study design** | **Associations (all taxonomic levels)** | **No. studies in review reporting differences** |
| --- | --- | --- | --- | --- |
| Food emulsifiers | SR [38] | 15 (Animal and in vitro human model) | 9 | 4 |
| Maltodextrin | SR [39] | 42 | 4 | 21 |
| CMC | PS [105] | 16; human intervention | 4 | N/A |
| CMC | PS [106] | 13; human faecal samples | 0 | N/A |
| P-80 | PS [106] | 13; human faecal samples | 3 | N/A |
| Maltodextrin | PS [106] | 13; human faecal samples | 3 | N/A |
| Dishwashing detergent | PS [106] | 13; human faecal samples | 10 | N/A |
| Carrageenan | PS [106] | 13; human faecal samples | 3 | N/A |
| Gum arabic | PS [108] | 51; human intervention | 3 | N/A |
| Xanthan gum | PS [109] | 60; human faecal samples (mouse model) | 2 | N/A |
| Preservatives | PS [110] | 3; human faecal sample donors | 4 | N/A |
| Sodium sulphite | PS [106] | 13; human faecal samples | 4 | N/A |
| Sodium benzoate | PS [106] | 13; human faecal samples | 2 | N/A |
| Nisin | PS [111] | Human colon model | 3 | N/A |
| Cinnamaldehyde | PS [106] | 13; human faecal samples | 2 | N/A |
| Aspartame | PS [106] | 13; human faecal samples | 4 | N/A |
| Aspartame | PS [112] | 31; human intervention | 0 (NS) | N/A |
| Aspartame | PS [113] | 22; human intervention | 0 (NS) | N/A |
| Aspartame | PS [114] | 30; human intervention | 0 (NS) | N/A |
| Trehalose | PS [116] | In vitro, lab study | 1 | N/A |
| Stevioside | PS [117] | In vitro model | 3 | N/A |
| Stevioside | PS [106] | 13; human faecal samples | 0 (NS) | N/A |
| Non caloric sweeteners | PS [118] | Human samples, animal model | 10 | N/A |
| Non caloric sweeteners | PS [119] | 120; human intervention | Dysbiosis | N/A |
| Non caloric sweeteners | PS [120] | 46; human intervention | 0 (NS) | N/A |
| Sugar alcohols | PS [122] | 40; human intervention | 4 | N/A |
| Sugar alcohols | PS [123] | 19; human intervention | 1 | N/A |
| Sugar alcohols | PS [124] | 75; human intervention | 3 | N/A |
| Sugar alcohols | PS [125] | 36; human intervention | 4 | N/A |
| Sugar alcohols | PS [126] | 47; human intervention | 3 | N/A |
| Sweeteners | PS [153] | 197 sample: in vitro | 7/8 altered metaproteome | N/A |
| Polydextrose | PS [128] | 33; human intervention | 1 | N/A |
| Titanium dioxide | PS [106] | In vitro, human stool | 1 | N/A |
| Allura red (in vitro) | PS [129] | In Vitro | 3 | N/A |
| Allura red (in vitro) | PS [130] | In Vitro | 3 | N/A |
| Sudan I,II,III,IV, Para red | PS [131] | Preclinical | 11 | N/A |
| Natural food chemicals | PS [132] | Gut simulator model | 4 | N/A |
| Oxalate | PS [133] | NR | 3 | N/A |
| mTG | PS [134] | NR | Dysbiosis | N/A |
| Monosodium glutamate | PS [152] | 12: human intervention | 0 (NS) | N/A |

mTG: Microbial transglutaminase, N/A: Not applicable, NaR: Narrative review, PS: primary study, SR: systematic review

**Supplementary Table 8: Associations between individual foods and microbiota reported in reviews and primary studies**

| **Dietary factor** | **Review or primary study** | **Number of studies (SR) or participants (PS) and study design** | **Associations (all taxonomic levels)** | **No. studies in review reporting differences** |
| --- | --- | --- | --- | --- |
| Nuts | SR [47] | 8 | 4 | 8 |
| Nuts | SR [48] | 8 | 10 | 8 |
| Walnuts | PS [143] | 18; human intervention | 8 | N/A |
| Walnuts | PS [146] | 27; human intervention | 6 | N/A |
| Walnuts | PS [165] | 194; human intervention | 5 | N/A |
| Almonds | PS [149] | 73; human intervention | 11 | N/A |
| Almonds | PS [144] | 18; human intervention | 9 | N/A |
| Hazelnuts | PS [147] | 30; human XC | 0 (NS) | N/A |
| Pistachios | PS [141] | 16; human intervention | 6 | N/A |
| Gin | SR [14] | 86 | 3 | 2 |
| Kombucha | SR [49] | 15 | 8 | 4 |
| Red wine | PS [151] | 10; human intervention | 4 \| 3 | N/A |
| Coffee | PS [54] | 1098; cross sectional | 1 | N/A |
| Fermented foods | PS [158] | 6811; human XC (repeat) | 12 | N/A |
| Fermented dairy | PS [142] | 1135; observational | 5 | N/A |
| Fermented dairy | PS [54] | 1098; cross sectional | 5 | N/A |
| Fermented foods | PS [159] | 18; human intervention | 9 | N/A |

N/A: Not applicable, NaR: Narrative review, PS: primary study, SR: systematic review
